# Supplementary figures and images for: Structure-based 3D-Pharmacophore modeling to discover novel interleukin 6 inhibitors: An in silico screening, molecular dynamics simulations and binding free energy calculations
Source: PLoS One. 2022 Apr 6;17(4):e0266632. doi: 10.1371/journal.pone.0266632 (PMC8986010; doi:10.1371/journal.pone.0266632)

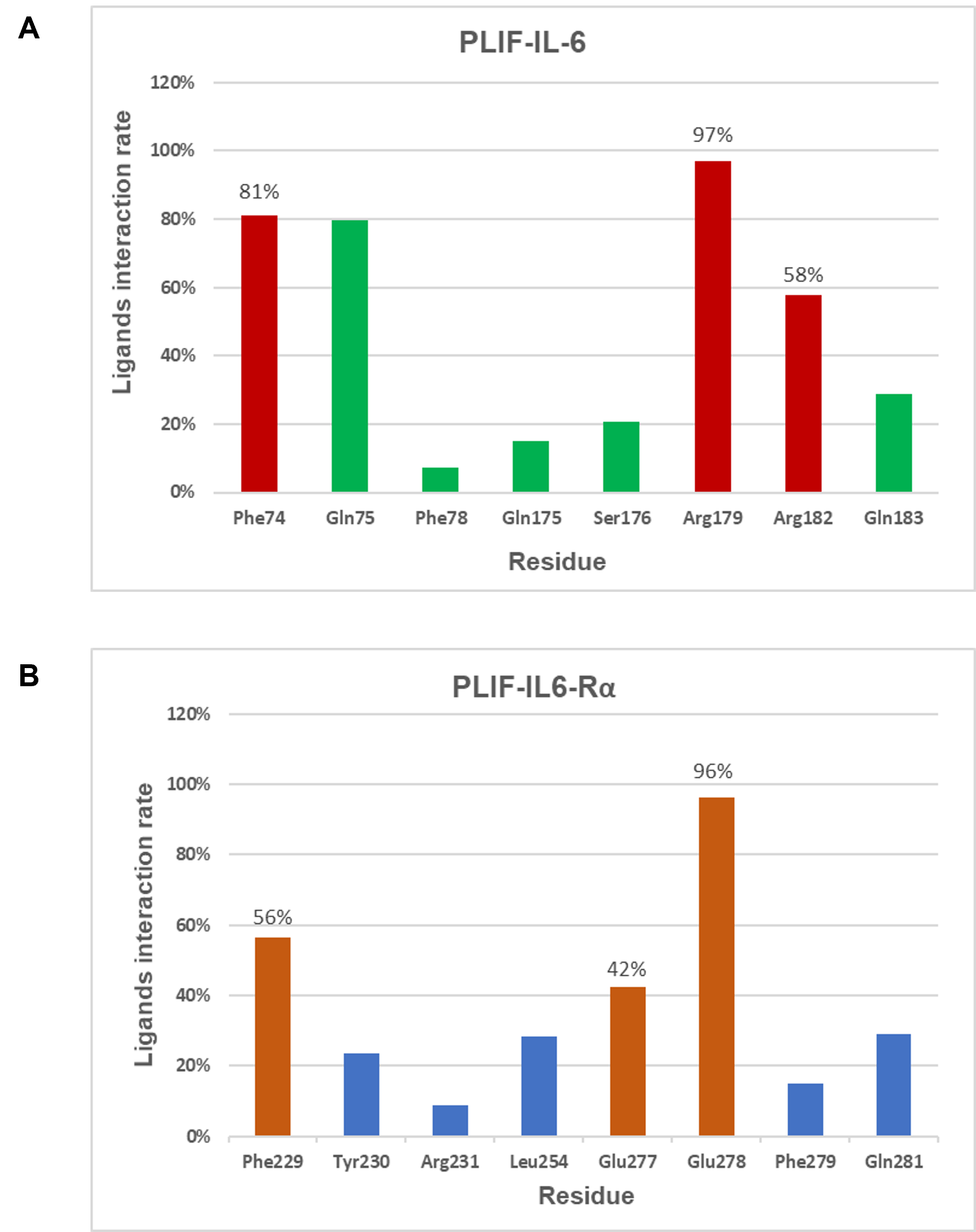

Supplement: S1 Fig — The interaction frequency of individual residue on IL-6 (A) and IL-6Rα (B) with the docking poses of ligands. (TIF) [file pone.0266632.s001.tif]

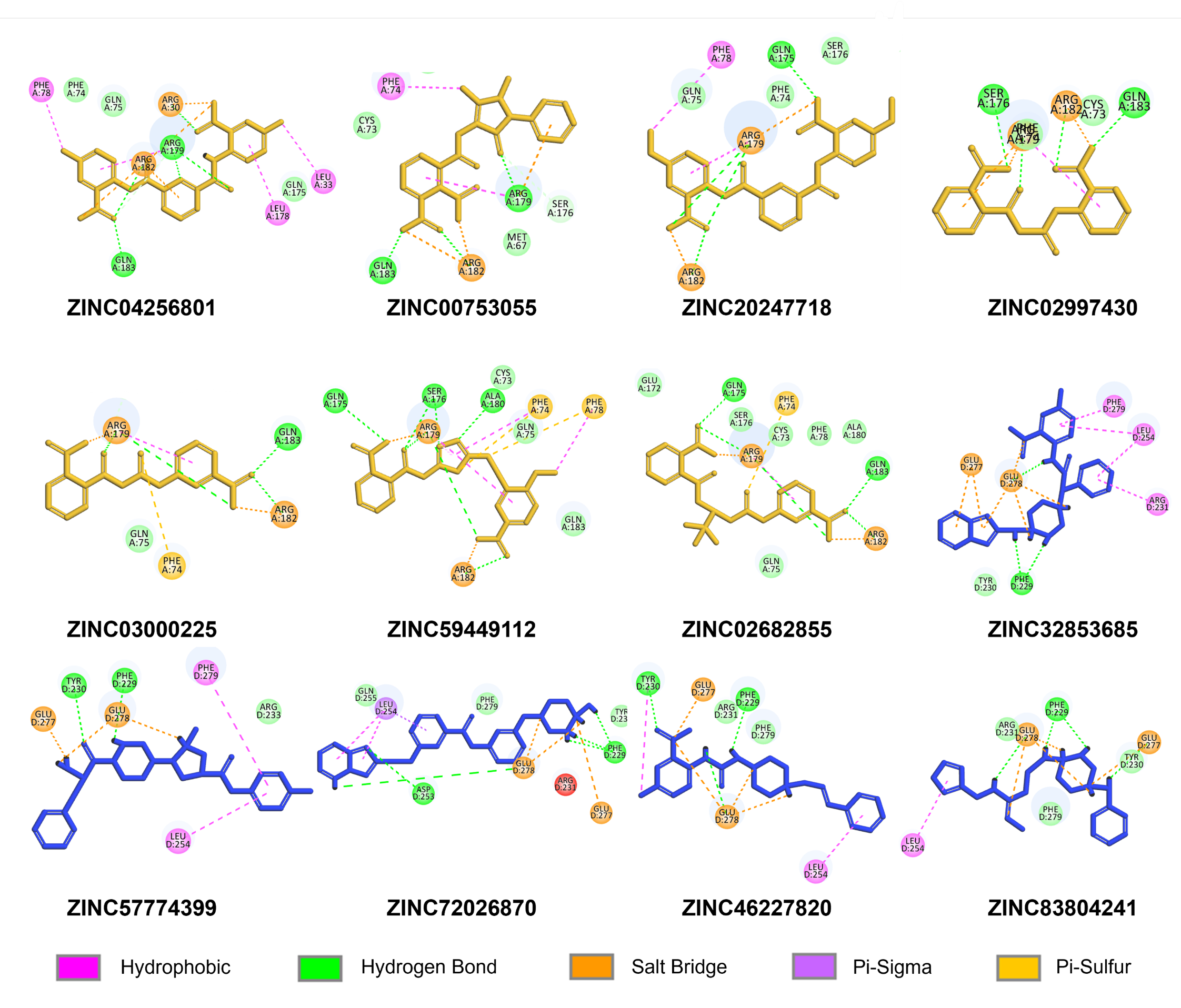

Supplement: S2 Fig — The yellow and blue ligands are potential compounds binding to IL-6 and IL-6Rα, respectively. (TIF) [file pone.0266632.s002.tif]

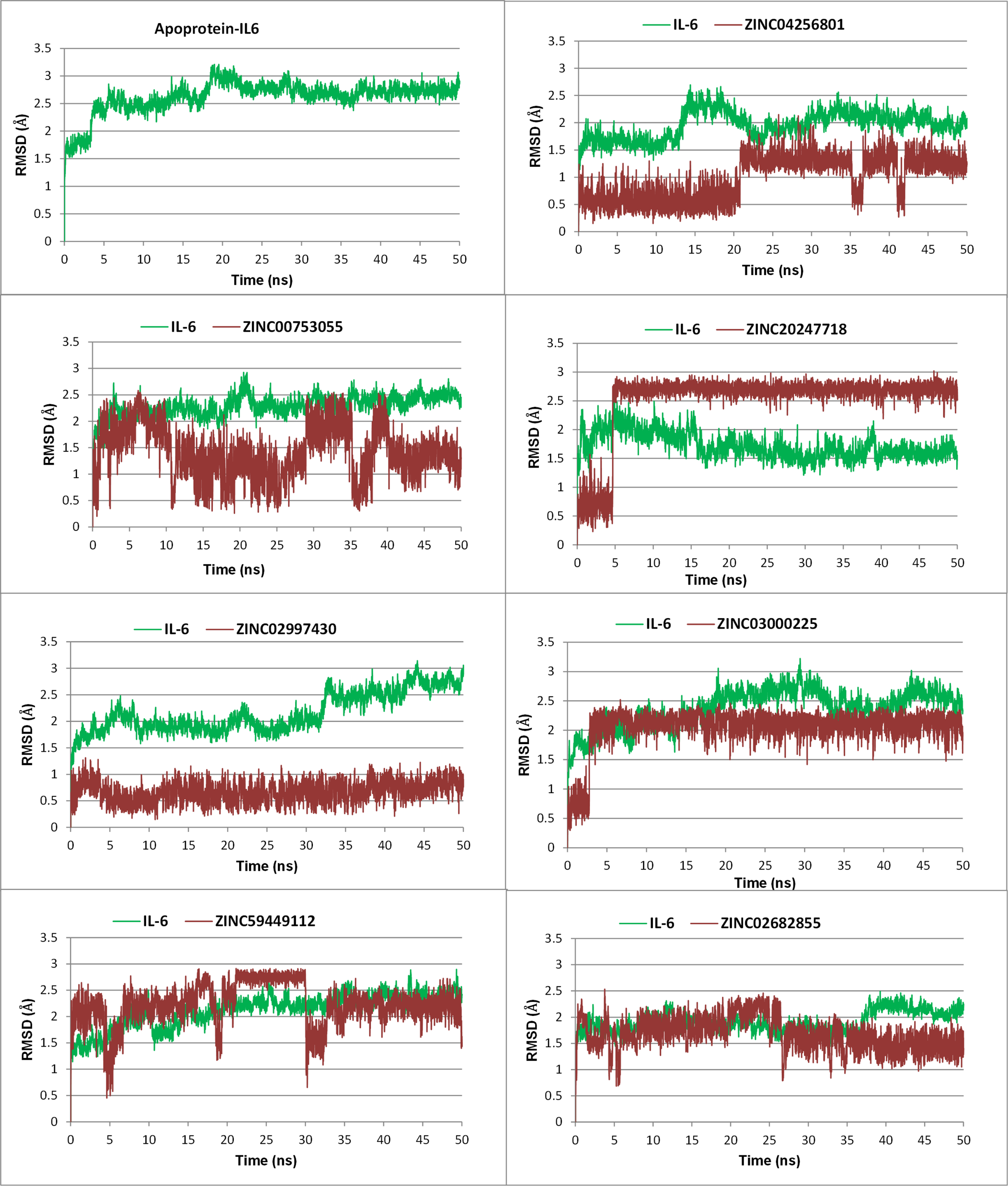

Supplement: S3 Fig — (TIF) [file pone.0266632.s003.tif]

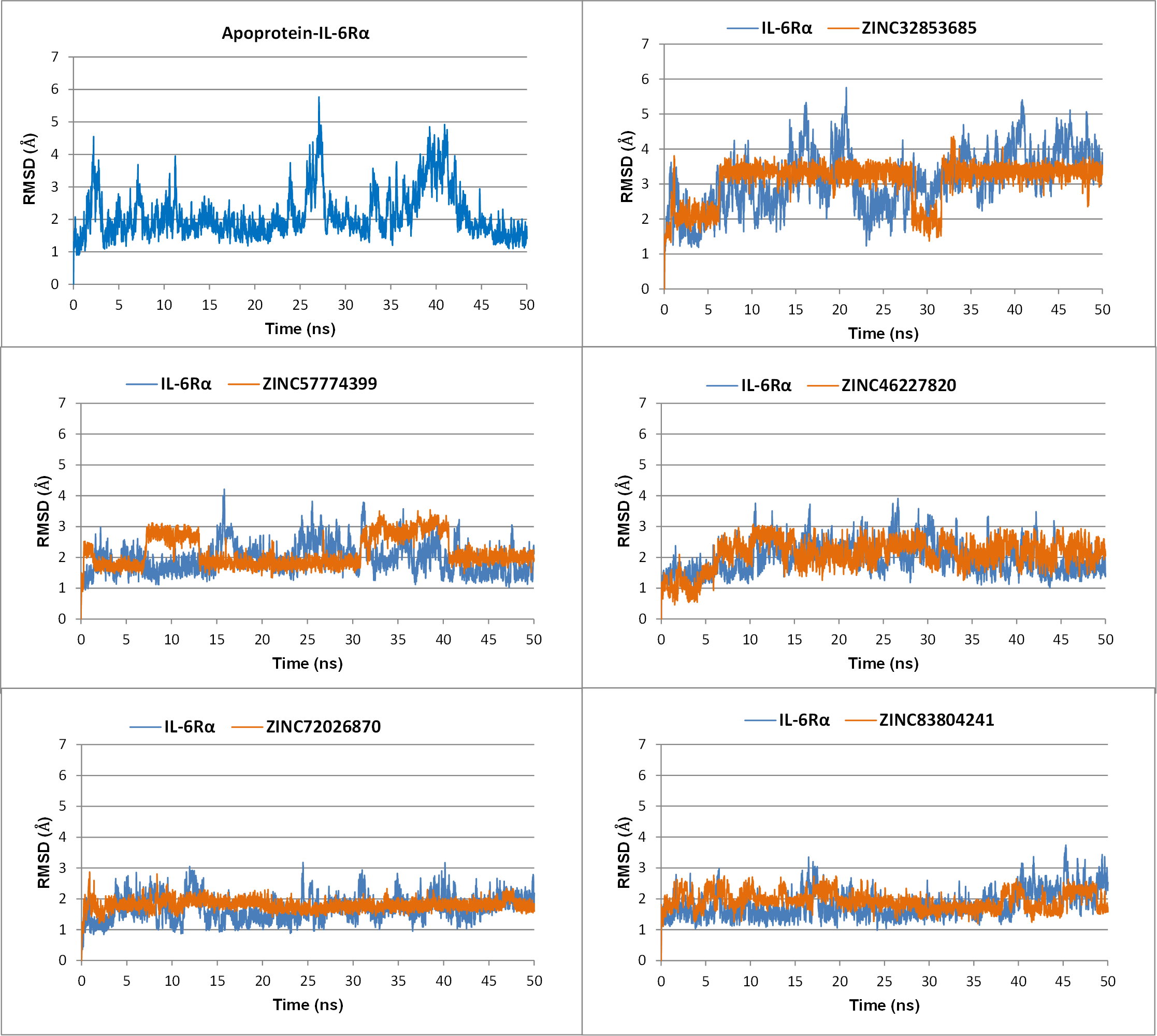

Supplement: S4 Fig — (TIF) [file pone.0266632.s004.tif]

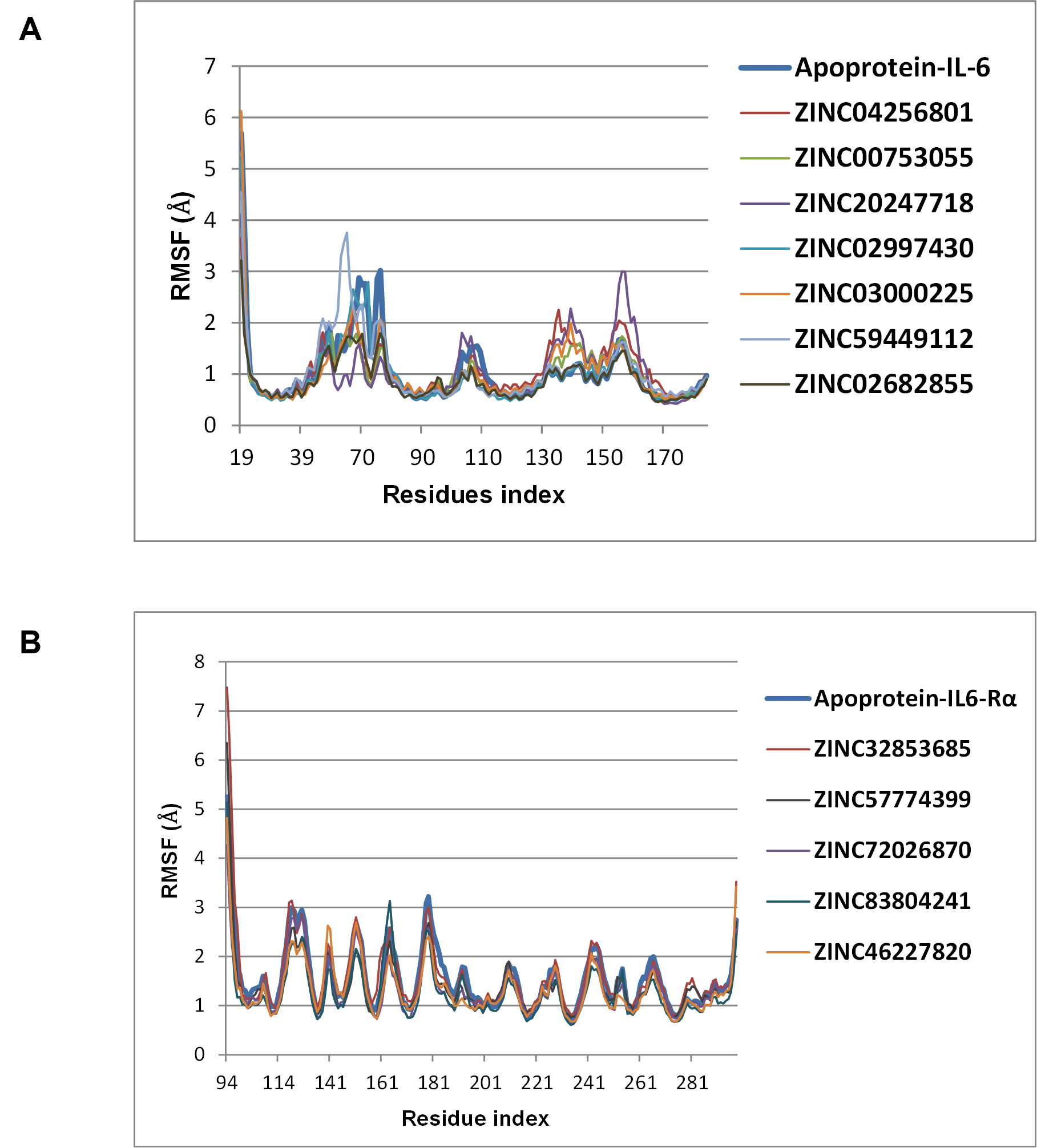

Supplement: S5 Fig — S4A Fig illustrated the RMSF Cα values of the IL-6 apoprotein and its complexes with 7 ligands, S4B Fig presented RMSF Cα values of the IL-6Rα apoprotein and its complexes with 5 ligands calculated by 50 ns MDs trajectories. (TIF) [file pone.0266632.s005.tif]

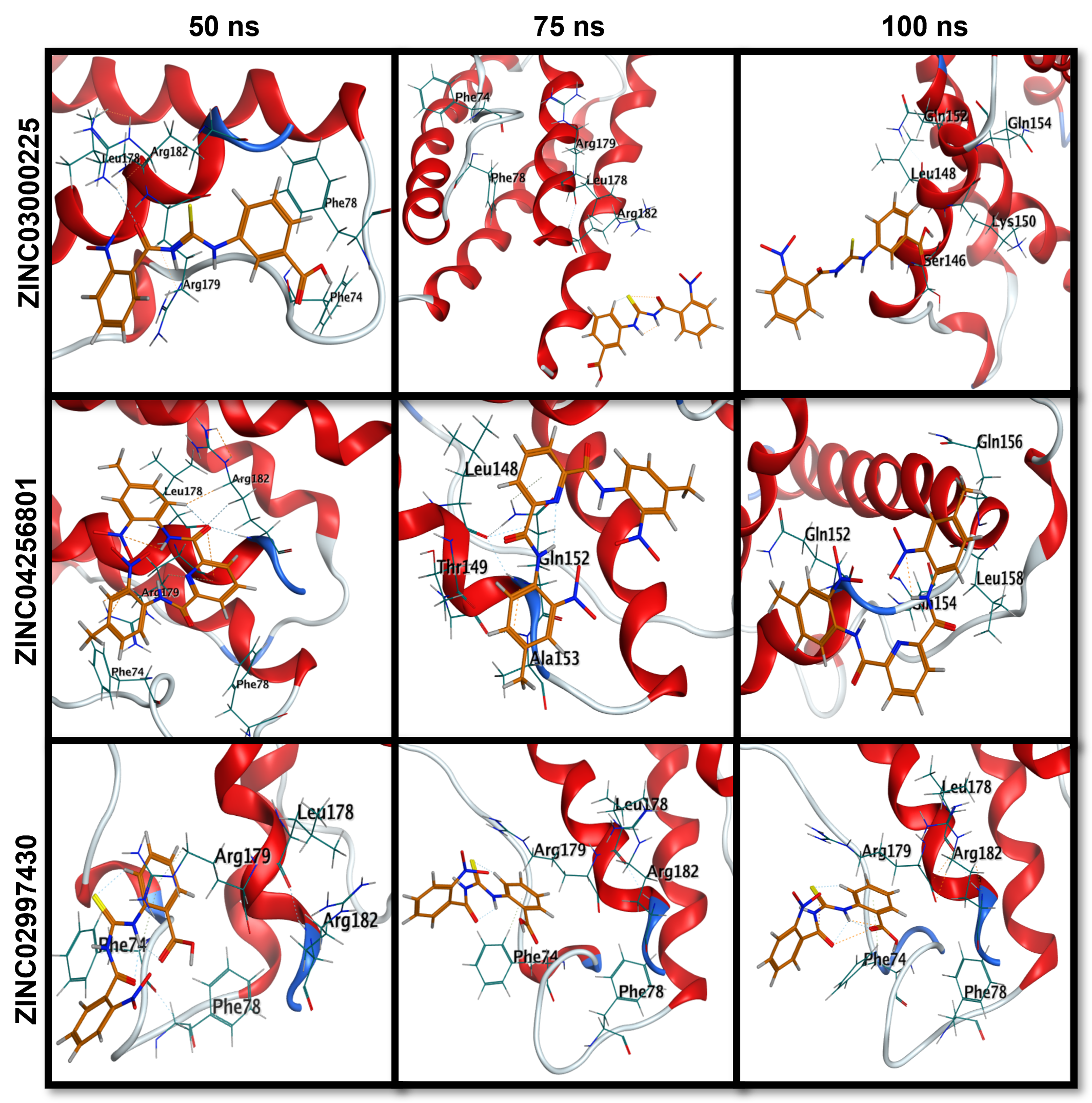

Supplement: S6 Fig — Protein-ligand conformations at every 25 ns of the last 50 ns MDs trajectories for ZINC03000225, ZINC04256801, ZINC02997430. (TIF) [file pone.0266632.s006.tif]

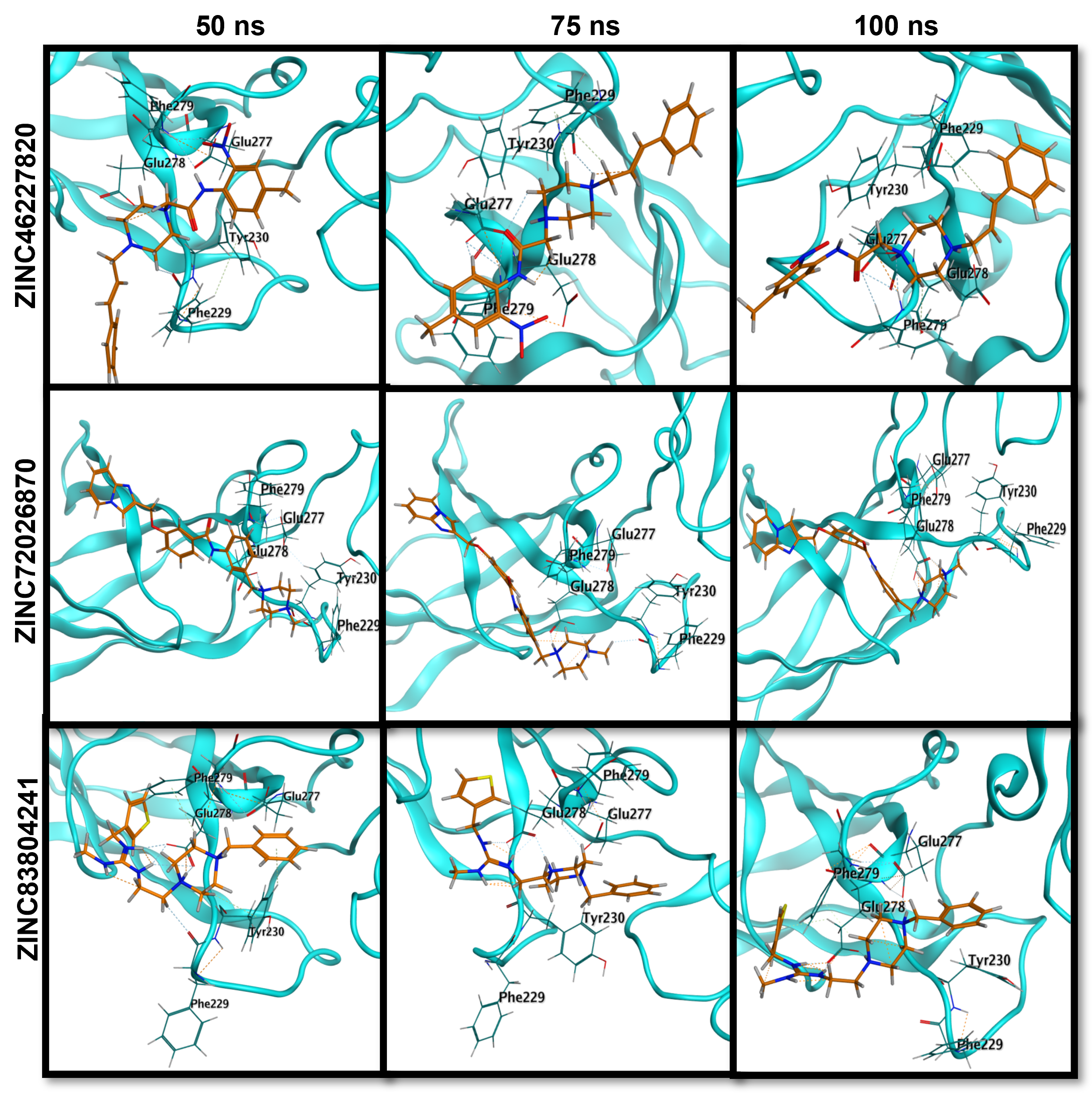

Supplement: S7 Fig — Protein-ligand conformations at every 25 ns of the last 50 ns MDs trajectories for ZINC46227820, ZINC72026870, ZINC83304241. (TIF) [file pone.0266632.s007.tif]

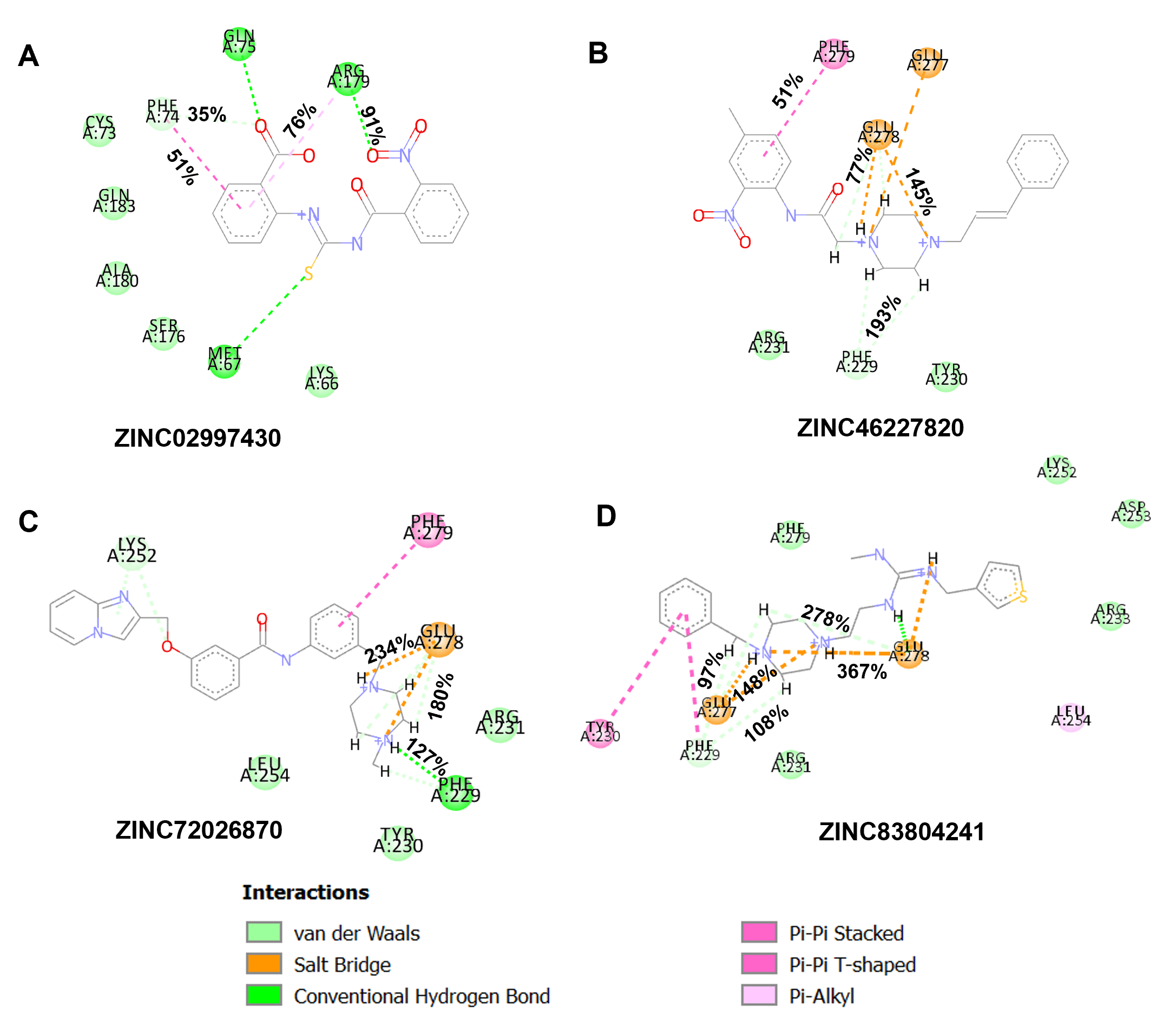

Supplement: S8 Fig — S8A-S8D Fig correspond to ZINC02997430, ZINC46227820, ZINC72026870, and ZINC83804241, respectively. (TIF) [file pone.0266632.s008.tif]
