# Supplementary material for: Structure-based 3D-Pharmacophore modeling to discover novel interleukin 6 inhibitors: An in silico screening, molecular dynamics simulations and binding free energy calculations
Source: PLoS One. 2022 Apr 6;17(4):e0266632. doi: 10.1371/journal.pone.0266632 (PMC8986010; doi:10.1371/journal.pone.0266632)
Supplement: S1 Table — (PDF) [file pone.0266632.s009.pdf]

S1 Table. The docking results of 235 potential IL-6 inhibitors

No	Name	Pharmacophore models	Docking Score (kJ/mol)
1	ZINC04256801	Ph_1	-27.57
2	ZINC00753055	Ph_1	-27.48
3	ZINC20247718	Ph_1	-27.06
4	ZINC01065668	Ph_1	-26.90
5	ZINC32853685	Ph_2	-26.44
6	ZINC57774399	Ph_2	-26.24
7	ZINC01032960	Ph_1	-26.19
8	ZINC04464767	Ph_1	-25.71
9	ZINC02997430	Ph_1	-25.64
10	ZINC05427861	Ph_1	-25.51
11	ZINC03000225	Ph_1	-25.43
12	ZINC09492626	Ph_1	-25.36
13	ZINC72026870	Ph_2	-25.31
14	ZINC46227820	Ph_2	-25.25
15	ZINC59449112	Ph_1	-25.20
16	ZINC83804241	Ph_2	-25.07
17	ZINC02682855	Ph_1	-25.00
18	ZINC16449272	Ph_1	-24.93
19	ZINC39388796	Ph_1	-24.92
20	ZINC06884691	Ph_1	-24.72
21	ZINC12807190	Ph_1	-24.64
22	ZINC71284970	Ph_1	-24.53
23	ZINC20614554	Ph_1	-24.51
24	ZINC02997569	Ph_1	-24.46
25	ZINC40148799	Ph_1	-24.20
26	ZINC59389339	Ph_2	-24.19
27	ZINC06615593	Ph_1	-24.04
28	ZINC25574579	Ph_1	-24.00
29	ZINC15667990	Ph_1	-23.91

30	ZINC40803750	Ph_1	-23.86
31	ZINC06884452	Ph_1	-23.82
32	ZINC01086137	Ph_1	-23.81
33	ZINC92648559	Ph_1	-23.71
34	ZINC95364161	Ph_1	-23.64
35	ZINC17087520	Ph_2	-23.60
36	ZINC20614427	Ph_1	-23.57
37	ZINC59449113	Ph_1	-23.53
38	ZINC25777839	Ph_2	-23.43
39	ZINC40374767	Ph_1	-23.40
40	ZINC60740730	Ph_1	-23.38
41	ZINC16449279	Ph_1	-23.35
42	ZINC02102008	Ph_1	-23.31
43	ZINC20075512	Ph_1	-23.26
44	ZINC15084912	Ph_1	-23.25
45	ZINC16449300	Ph_1	-23.25
46	ZINC06088935	Ph_1	-23.24
47	ZINC04551189	Ph_1	-23.22
48	ZINC00456087	Ph_1	-23.21
49	ZINC00513306	Ph_1	-23.13
50	ZINC82107635	Ph_2	-23.05
51	ZINC19793889	Ph_1	-23.02
52	ZINC04013419	Ph_1	-23.01
53	ZINC02828852	Ph_1	-22.99
54	ZINC15953299	Ph_1	-22.81
55	ZINC02213344	Ph_1	-22.81
56	ZINC59854382	Ph_1	-22.75
57	ZINC04044908	Ph_1	-22.73
58	ZINC91686816	Ph_1	-22.73
59	ZINC71288325	Ph_1	-22.67
60	ZINC04538714	Ph_1	-22.67
61	ZINC03000938	Ph_1	-22.63

62	ZINC57140875	Ph_1	-22.62
63	ZINC78997274	Ph_1	-22.59
64	ZINC63444522	Ph_1	-22.52
65	ZINC21004470	Ph_1	-22.51
66	ZINC92319961	Ph_1	-22.50
67	ZINC06668580	Ph_1	-22.50
68	ZINC36734323	Ph_2	-22.49
69	ZINC71285013	Ph_1	-22.49
70	ZINC89642414	Ph_1	-22.45
71	ZINC14006045	Ph_1	-22.43
72	ZINC05568418	Ph_1	-22.38
73	ZINC59487809	Ph_1	-22.37
74	ZINC16449295	Ph_1	-22.37
75	ZINC02342223	Ph_1	-22.32
76	ZINC89642180	Ph_1	-22.26
77	ZINC17146632	Ph_1	-22.24
78	ZINC05034528	Ph_1	-22.23
79	ZINC05595304	Ph_1	-22.23
80	ZINC38605693	Ph_2	-22.22
81	ZINC78629623	Ph_1	-22.15
82	ZINC04713201	Ph_1	-22.15
83	ZINC32962119	Ph_1	-22.14
84	ZINC31160070	Ph_1	-22.12
85	ZINC33363057	Ph_1	-22.10
86	ZINC04526151	Ph_1	-22.09
87	ZINC70503642	Ph_1	-22.08
88	ZINC66810410	Ph_1	-22.05
89	ZINC89642254	Ph_1	-22.03
90	ZINC92646196	Ph_1	-22.01
91	ZINC07007223	Ph_1	-22.00
92	ZINC04394123	Ph_1	-21.96
93	ZINC88700488	Ph_1	-21.87

94	ZINC05130339	Ph_1	-21.85
95	ZINC78783778	Ph_1	-21.81
96	ZINC04093678	Ph_1	-21.81
97	ZINC09119843	Ph_1	-21.79
98	ZINC83139305	Ph_1	-21.78
99	ZINC06571299	Ph_1	-21.76
100	ZINC08190089	Ph_1	-21.70
101	ZINC57838157	Ph_2	-21.69
102	ZINC95412711	Ph_1	-21.67
103	ZINC00096720	Ph_1	-21.66
104	ZINC07780460	Ph_1	-21.64
105	ZINC15953254	Ph_1	-21.62
106	ZINC25576152	Ph_1	-21.61
107	ZINC10962685	Ph_1	-21.57
108	ZINC19142785	Ph_1	-21.53
109	ZINC66146080	Ph_1	-21.49
110	ZINC65867112	Ph_1	-21.47
111	ZINC66868285	Ph_1	-21.45
112	ZINC95362397	Ph_1	-21.44
113	ZINC03050549	Ph_1	-21.41
114	ZINC00130543	Ph_1	-21.41
115	ZINC04871932	Ph_1	-21.37
116	ZINC01030873	Ph_1	-21.35
117	ZINC92360672	Ph_1	-21.34
118	ZINC21157350	Ph_1	-21.32
119	ZINC00513304	Ph_1	-21.30
120	ZINC04307500	Ph_1	-21.30
121	ZINC19839589	Ph_2	-21.29
122	ZINC32101606	Ph_1	-21.27
123	ZINC24543620	Ph_1	-21.25
124	ZINC62022798	Ph_1	-21.24
125	ZINC13642337	Ph_1	-21.24

126	ZINC04578300	Ph_1	-21.21
127	ZINC38186317	Ph_1	-21.21
128	ZINC06445812	Ph_1	-21.20
129	ZINC78632635	Ph_1	-21.18
130	ZINC58835024	Ph_1	-21.17
131	ZINC66866608	Ph_1	-21.16
132	ZINC89642200	Ph_1	-21.15
133	ZINC59219285	Ph_1	-21.14
134	ZINC02421992	Ph_1	-21.14
135	ZINC64896875	Ph_1	-21.12
136	ZINC54042265	Ph_1	-21.10
137	ZINC06343831	Ph_1	-21.06
138	ZINC02604054	Ph_1	-21.04
139	ZINC06393328	Ph_1	-21.03
140	ZINC05293200	Ph_1	-21.02
141	ZINC67868429	Ph_1	-21.00
142	ZINC59294092	Ph_1	-20.99
143	ZINC38614234	Ph_1	-20.99
144	ZINC12785726	Ph_1	-20.97
145	ZINC04324710	Ph_1	-20.97
146	ZINC04507720	Ph_1	-20.95
147	ZINC71280183	Ph_1	-20.95
148	ZINC00821875	Ph_1	-20.93
149	ZINC20614484	Ph_1	-20.92
150	ZINC83138269	Ph_1	-20.92
151	ZINC20614745	Ph_1	-20.92
152	ZINC16248184	Ph_1	-20.92
153	ZINC60741448	Ph_1	-20.90
154	ZINC89642243	Ph_1	-20.90
155	ZINC38604138	Ph_2	-20.90
156	ZINC05610856	Ph_1	-20.86
157	ZINC83291266	Ph_1	-20.84

158	ZINC06884715	Ph_1	-20.82
159	ZINC08146024	Ph_1	-20.81
160	ZINC09544466	Ph_1	-20.80
161	ZINC80719235	Ph_1	-20.77
162	ZINC21454018	Ph_1	-20.76
163	ZINC12340662	Ph_1	-20.76
164	ZINC00235141	Ph_1	-20.73
165	ZINC38605570	Ph_2	-20.72
166	ZINC05293221	Ph_1	-20.71
167	ZINC03305881	Ph_1	-20.71
168	ZINC45343469	Ph_2	-20.68
169	ZINC42756629	Ph_1	-20.66
170	ZINC45313992	Ph_2	-20.65
171	ZINC09597043	Ph_1	-20.65
172	ZINC06884498	Ph_1	-20.64
173	ZINC06343832	Ph_1	-20.63
174	ZINC78653947	Ph_1	-20.62
175	ZINC00806582	Ph_1	-20.62
176	ZINC12249905	Ph_1	-20.62
177	ZINC67169616	Ph_1	-20.58
178	ZINC38932493	Ph_1	-20.56
179	ZINC12040472	Ph_1	-20.56
180	ZINC83326401	Ph_1	-20.55
181	ZINC76881380	Ph_1	-20.55
182	ZINC64479409	Ph_1	-20.53
183	ZINC16498138	Ph_1	-20.51
184	ZINC18162160	Ph_1	-20.51
185	ZINC12883677	Ph_2	-20.49
186	ZINC20494770	Ph_1	-20.49
187	ZINC62165059	Ph_1	-20.47
188	ZINC36354526	Ph_1	-20.47
189	ZINC92868592	Ph_2	-20.46

190	ZINC02555634	Ph_1	-20.43
191	ZINC06444524	Ph_1	-20.43
192	ZINC13596907	Ph_2	-20.42
193	ZINC93147393	Ph_2	-20.41
194	ZINC83142456	Ph_1	-20.41
195	ZINC36366663	Ph_1	-20.38
196	ZINC33762919	Ph_1	-20.37
197	ZINC01879821	Ph_1	-20.36
198	ZINC13151931	Ph_1	-20.34
199	ZINC00943134	Ph_1	-20.34
200	ZINC59328624	Ph_1	-20.34
201	ZINC21991919	Ph_1	-20.33
202	ZINC76092798	Ph_1	-20.32
203	ZINC14932863	Ph_2	-20.31
204	ZINC15134528	Ph_1	-20.31
205	ZINC16215148	Ph_1	-20.30
206	ZINC66200569	Ph_1	-20.30
207	ZINC19315743	Ph_1	-20.28
208	ZINC04460154	Ph_1	-20.27
209	ZINC09368301	Ph_1	-20.26
210	ZINC78995293	Ph_1	-20.26
211	ZINC71284969	Ph_1	-20.24
212	ZINC49244904	Ph_1	-20.23
213	ZINC19456797	Ph_2	-20.21
214	ZINC72425018	Ph_1	-20.21
215	ZINC71286399	Ph_1	-20.19
216	ZINC06615810	Ph_1	-20.19
217	ZINC67818954	Ph_1	-20.15
218	ZINC27825544	Ph_1	-20.13
219	ZINC36354062	Ph_1	-20.12
220	ZINC16450526	Ph_1	-20.12
221	ZINC72470674	Ph_1	-20.12

222	ZINC06884501	Ph_1	-20.11
223	ZINC04456230	Ph_1	-20.09
224	ZINC06552344	Ph_1	-20.08
225	ZINC71279249	Ph_1	-20.08
226	ZINC02690589	Ph_1	-20.08
227	ZINC06846302	Ph_1	-20.07
228	ZINC18201322	Ph_1	-20.05
229	ZINC04990683	Ph_1	-20.04
230	ZINC25066903	Ph_1	-20.04
231	ZINC16450528	Ph_1	-20.03
232	ZINC36075322	Ph_1	-20.03
233	ZINC04635743	Ph_1	-20.02
234	ZINC04280971	Ph_1	-20.01
235	ZINC72191477	Ph_1	-20.01
