# Supplementary material for: Structure-based 3D-Pharmacophore modeling to discover novel interleukin 6 inhibitors: An in silico screening, molecular dynamics simulations and binding free energy calculations
Source: PLoS One. 2022 Apr 6;17(4):e0266632. doi: 10.1371/journal.pone.0266632 (PMC8986010; doi:10.1371/journal.pone.0266632)
Supplement: S2 Table — (PDF) [file pone.0266632.s010.pdf]

S2 Table. The chemical structures and properties of potential IL-6/IL-6Rα binders

No.	Name	Structure	Properties (Lipinski's results)
**1**	ZINC04256801 ($C_{21}H_{17}N_5O_6$)	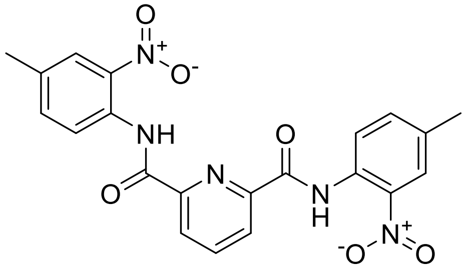	MW: 435.40<br>LogP: 4.02<br>HBA: 7<br>HBD: 2
**2**	ZINC00753055 ($C_{19}H_{16}N_4O_6$)	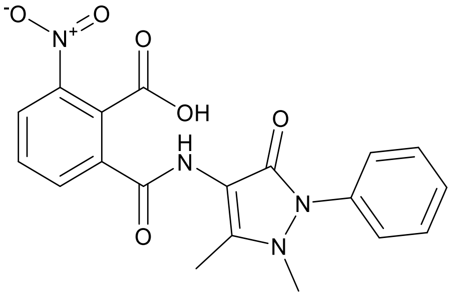	MW: 396.36<br>LogP: 2.34<br>HBA: 8<br>HBD: 1
**3**	ZINC20247718 ($C_{21}H_{17}N_5O_8$)	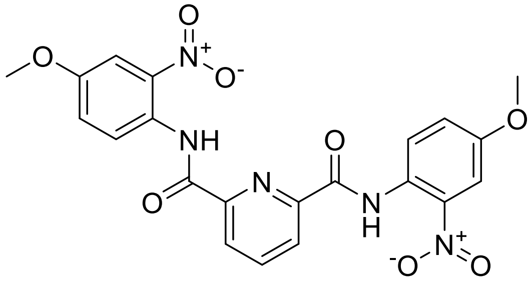	MW: 467.39<br>LogP: 3.42<br>HBA: 9<br>HBD: 2
**4**	ZINC02997430 ($C_{15}H_{11}N_3O_5S$)	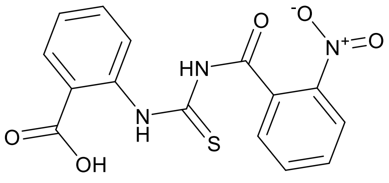	MW: 345.34<br>LogP: 2.42<br>HBA: 6<br>HBD: 2
**5**	ZINC03000225 ($C_{15}H_{11}N_3O_5S$)	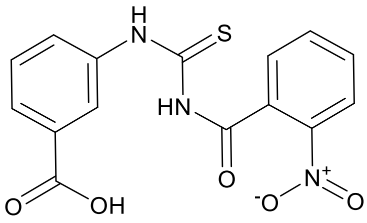	MW: 345.33<br>LogP: 2.42<br>HBA: 6<br>HBD: 2

**6**	ZINC59449112 ($C_{18}H_{14}N_4O_6S_2$)	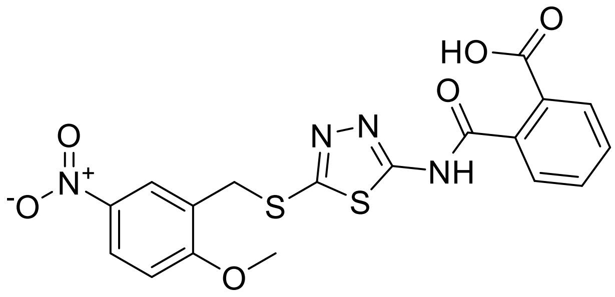	MW: 446.47<br>LogP: 3.7<br>HBA: 8<br>HBD: 2
**7**	ZINC02682855 ($C_{17}H_{13}Cl_3N_4O_5S$)	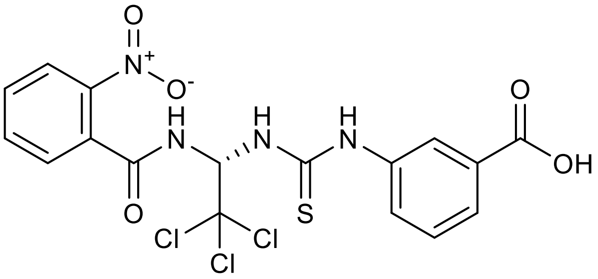	MW: 491.74<br>LogP: 3.71<br>HBA: 6<br>HBD: 3
**8**	ZINC32853685 ($C_{27}H_{28}N_6O_3$)	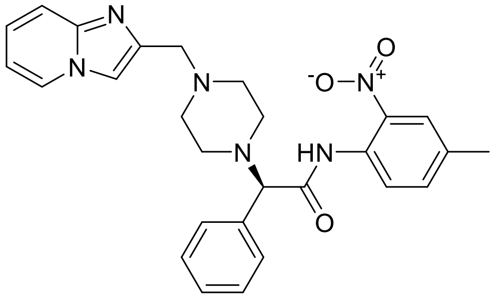	MW: 484.56<br>LogP: 4.05<br>HBA: 7<br>HBD: 1
**9**	ZINC72026870 ($C_{27}H_{29}N_5O_2$)	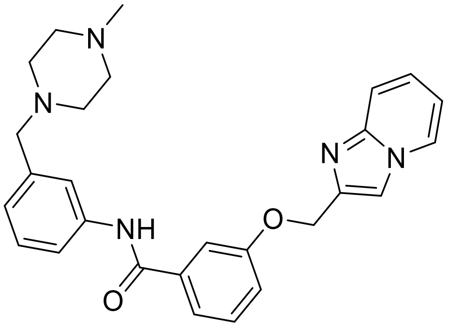	MW: 455.56<br>LogP: 3.91<br>HBA: 7<br>HBD: 1
**10**	ZINC46227820 ($C_{22}H_{26}N_4O_3$)	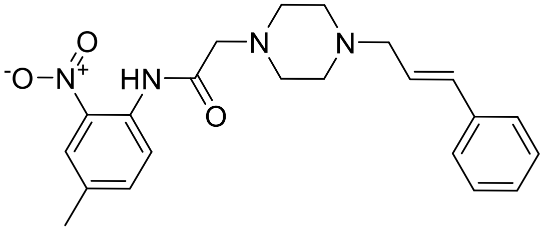	MW: 394.48<br>LogP: 3.17<br>HBA: 7<br>HBD: 1

**11**	ZINC57774399 ($C_{26}H_{33}FN_4O_2$)	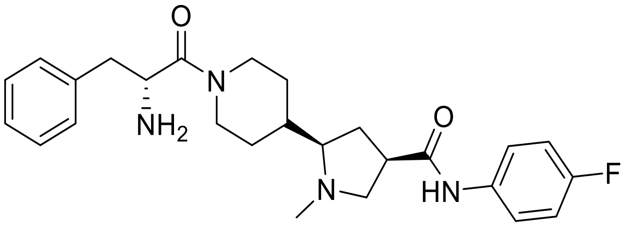	MW: 452.57<br>LogP: 2.89<br>HBA: 2<br>HBD: 3
**12**	ZINC83804241 ($C_{20}H_{29}N_5S$)	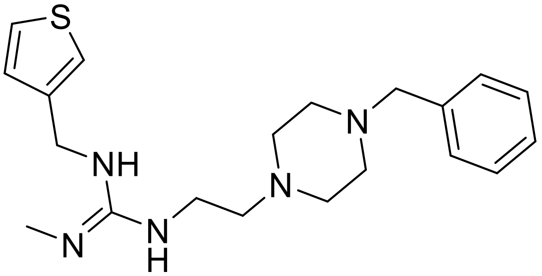	MW: 371.56<br>LogP: 2.23<br>HBA: 3<br>HBD: 2

MW: Molecular weight; LogP: Octanol-water partition coefficient; HBA: Hydrogen bond acceptor; HBD: Hydrogen bond donor.
